# Sustained Hypoxia Suppresses Joint Destruction in a Rat Model of Rheumatoid Arthritis via Negative Feedback of Hypoxia Inducible Factor-1α

**DOI:** 10.3390/ijms22083898

**Published:** 2021-04-09

**Authors:** Kenta Kaihara, Shuji Nakagawa, Yuji Arai, Hiroaki Inoue, Shinji Tsuchida, Yuta Fujii, Yoichiro Kamada, Tsunao Kishida, Osam Mazda, Kenji Takahashi

**Affiliations:** 1 Department of Orthopaedics, Graduate School of Medical Science, Kyoto Prefectural University of Medicine, Kawaramachi-Hirokoji, Kamigyo-ku, Kyoto 602-8566, Japan; kaihara5@koto.kpu-m.ac.jp (K.K.); hinoue@koto.kpu-m.ac.jp (H.I.); tuchi-kf@koto.kpu-m.ac.jp (S.T.); y-fujii@koto.kpu-m.ac.jp (Y.F.); kamada-y@koto.kpu-m.ac.jp (Y.K.); kenji-am@koto.kpu-m.ac.jp (K.T.); 2 Department of Sports and Para-Sports Medicine, Graduate School of Medical Science, Kyoto Prefectural University of Medicine, Kawaramachi-Hirokoji, Kamigyo-ku, Kyoto 602-8566, Japan; shushi@koto.kpu-m.ac.jp; 3 Department of Immunology, Graduate School of Medical Science, Kyoto Prefectural University of Medicine, Kawaramachi-Hirokoji, Kamigyo-ku, Kyoto 602-8566, Japan; tsunao@koto.kpu-m.ac.jp (T.K.); mazda@koto.kpu-m.ac.jp (O.M.)

**Keywords:** rheumatoid arthritis, hypoxia, HIF-1 alpha

## Abstract

Hypoxia inducible factor (HIF)-1α has been implicated in the pathogenesis of rheumatoid arthritis (RA). HIF-1α, which is expressed in hypoxia, is reversely suppressed in sustained hypoxia. Here, we investigated the inhibitory effect of hypoxia on arthritis by controlling HIF-1α. Rheumatoid fibroblast-like synoviocyte MH7A cells were cultured in a hypoxic incubator for up to 72 h to evaluate the expression of HIF-1. Furthermore, collagen-induced arthritis (CIA) model rats were maintained under 12% hypoxia in a hypoxic chamber for 28 days to evaluate the effect on arthritis. In MH7A cells, HIF-1α protein level increased at 3 h, peaked at 6 h, and subsequently decreased in a time-dependent manner. The transcription of pro-inflammatory cytokines increased at 1 h; however, they decreased after 3 h (*p* < 0.05). Deferoxamine-mediated activation of HIF-1α abolished the inhibitory effect of sustained hypoxia on pro-inflammatory cytokines. In the rat CIA model, the onset of joint swelling was delayed and arthritis was suppressed in the hypoxia group compared with the normoxia group (*p* < 0.05). Histologically, joint destruction was suppressed primarily in the cartilage. Thus, sustained hypoxia may represent a new safe, and potent therapeutic approach for high-risk patients with RA by suppressing HIF-1α expression.

## 1. Introduction

Rheumatoid arthritis (RA) impairs joint function because of joint destruction due to synovitis. Recently, the widespread use of biological agents against pro-inflammatory cytokines has enabled suppression of joint destruction, although the effect is insufficient in certain cases [1]. In addition, the use of biological agents results in side effects, such as infections and allergies, as well as economic burden [2,3]; therefore, a different approach for treating RA is required [4]. Compared with normal joints, RA-affected joint cavities are hypoxic due to inflammation [5]. Hypoxia inducible factor (HIF)-1α has been implicated in the cellular response to hypoxic stress. HIF-1α is continuously inactivated by prolyl hydroxylase domain-containing protein (PHD) under normoxic conditions [6]. PHD2 is encoded by the gene *EGLN1*. In case of a rapid change from normoxia to hypoxia, HIF-1α does not undergo PHD-mediated hydroxylation and is thus stable [7]. HIF-1α enhances the expression of pro-inflammatory cytokines, such as tumor necrosis factor (TNF)-α, via Toll-like receptors in rat-derived microglia [8]. In addition, HIF-1α activates its target gene, *VEGF*, which promotes angiogenesis and causes synovitis. Thus, HIF-1α is considered a key regulator of inflammation. Reports showed that drug-mediated inhibition of HIF-1α exerts therapeutic effects in other inflammatory diseases [9]. However, HIF-1α can be inhibited by drugs as well as by controlling oxygen concentration. Normally, the activity of HIF-1α is enhanced under short-term hypoxic conditions. However, Uchida et al. reported that under sustained hypoxia, HIF-1α is degraded by PHD, which is activated by negative feedback, resulting in a decrease in its activity [10]. Therefore, we focused on the finding that HIF-1α expression varies depending on the method of exposure to hypoxia. We hypothesized that the regulation of HIF-1α expression via sustained hypoxia might be a new therapeutic approach for RA. With this background, the purpose of this study was to evaluate the effects of sustained hypoxia on inflammation in MH7A cells and a rat model of collagen-induced arthritis (CIA) and assess this novel approach of hypoxia for arthritis control.

## 2. Results

### 2.1. Effects of Sustained Hypoxia on HIF-1α in MH7A Cells

**Figure 1:**
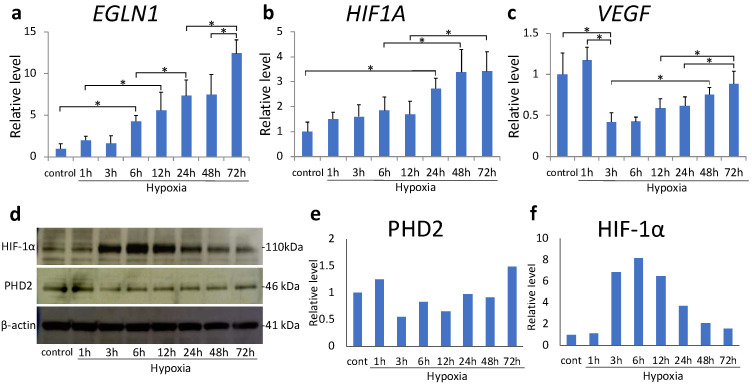
(**a**–**c**) *EGLN1* (**a**), hypoxia inducible factor (HIF) -1α (**b**), and *VEGF* (**c**) mRNA levels in MH7A cells were analyzed using quantitative reverse-transcription polymerase chain reaction (RT–PCR) after 72 h of hypoxia (1% O_2_). Each value represents the mean ± SD. (n = 6); * *p* < 0.05. (**d**–**f**) Prolyl hydroxylase domain-containing protein 2 (PHD2) and HIF-1α protein expression was analyzed using Western blotting; β-actin was used as the loading control.

We examined the effect of sustained hypoxia on HIF-1α expression in MH7A cells in response to hypoxia treatment. The expression of *EGLN1* increased in a time-dependent manner after 6 h (*p* = 0.012, Figure 1a). Similarly, *HIF1A* transcription significantly increased after 24 h (*p* < 0.001, Figure 1b); however, it plateaued after 48 h. Alternatively, *VEGF* was downregulated at 3 h (*p* < 0.001, Figure 1c) and subsequently became upregulated at 48 h compared with at 3 h (*p* = 0.005). The expression of PHD2 did not exhibit a consistent trend during hypoxic exposure, with no significant changes noted at any time point (Figure 1d,e). The level of HIF-1α protein increased after 3 h, peaked at 6 h, and subsequently decreased in a time-dependent manner (Figure 1d,f).

**Figure 2:**
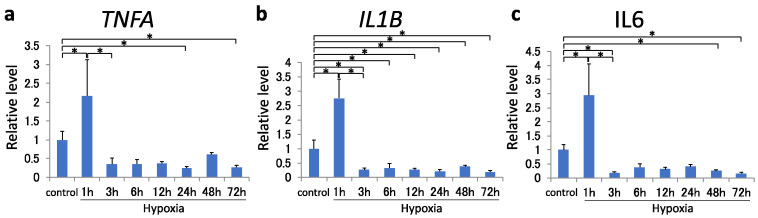
(**a**–**c**) *TNFA* (**a**), *IL1B* (**b**), and *IL6* (**c**) mRNA levels in MH7A cells were analyzed using quantitative reverse-transcription polymerase chain reaction after 72 h of hypoxia (1% O_2_). Each value represents the mean ± SD (n = 4); * *p* < 0.05.

mRNA expression of pro-inflammatory cytokines *TNFA*, *IL1B*, and *IL6* increased at 1 h (*p* < 0.001); however, their expression consistently decreased beginning at 3 h of hypoxic exposure (Figure 2a–c). Compared with the control, *TNFA* expression was suppressed at 24 and 72 h, *IL1B* expression was suppressed at all times after 3 h, and *IL6* expression was suppressed at 3, 48, and 72 h.

### 2.2. Effects of Deferoxamine (DFX) on HIF-1α after Sustained Hypoxia

**Figure 3:**
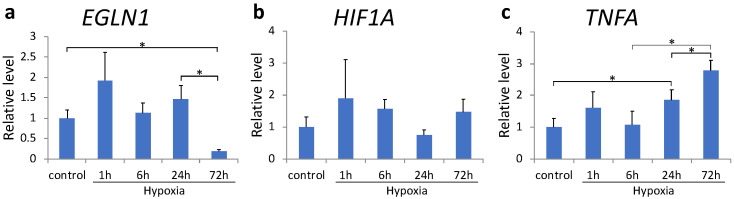
(**a**–**c**) *EGLN1* (**a**), *HIF1A* (**b**), and *TNFA* (**c**) mRNA expression in MH7A cells was analyzed using quantitative reverse-transcription polymerase chain reaction after culturing under hypoxia (1% O_2_) in the presence of deferoxamine. Each value represents the mean triplicate ± S.D. (n = 4); * *p* < 0.05.

The expression of pro-inflammatory cytokines decreased after 3 h of sustained hypoxia. To understand whether this effect was mediated by HIF-1α, we examined the effect of DFX, a PHD inhibitor, in MH7A cells. DFX treatment maximally reduced *EGLN1* expression at 72 h (*p* = 0.050, Figure 3a); however, no significant difference was observed in *HIF1A* expression (Figure 3b). *TNFA* transcription was most significantly upregulated 72 h after sustained hypoxia (*p* < 0.001, Figure 3c).

### 2.3. Effects of Sustained Hypoxia on Arthritis

**Figure 4:**
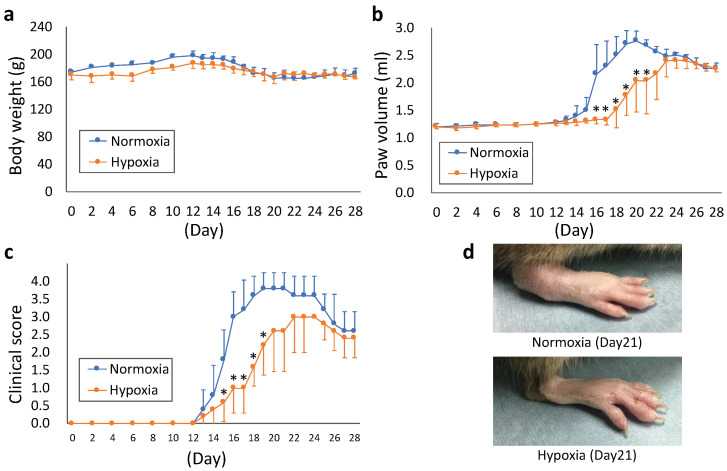
(**a**–**c**) Kinetic changes in body weight (**a**), paw volume (**b**), and clinical score (**c**) after immunization. The parameters were measured once every two days until day 12 and every day thereafter. (**d**) Representative images of arthrosis on day 21. Each value represents the mean ± SD (n = 5); * *p* < 0.05.

The rat model of CIA was maintained in a hypoxic chamber under 12% O_2_ hypoxia to evaluate hypoxia’s effect on arthritis. Body weight did not differ significantly between the normoxic and hypoxic groups during the entire observation period (from day 0 to 28, Figure 4a). In the normoxia group, the paw volume began increasing on day 14, whereas in the hypoxia group, an increase in paw volume was not observed until day 18 (Figure 4b), indicating a delay in the appearance of joint swelling. Compared with the normoxia group, the paw volume decreased significantly in the hypoxia group from days 16 to 21 (*p* < 0.05). Although the clinical score increased from day 13 in both groups (Figure 4c), it was significantly lower in the hypoxia group than in the normoxia group from days 15 to 19 (*p* < 0.05). The representative images of joint swelling on day 21 are shown in Figure 4d.

### 2.4. Histological Effects of Sustained Hypoxia on Arthritis

**Figure 5:**
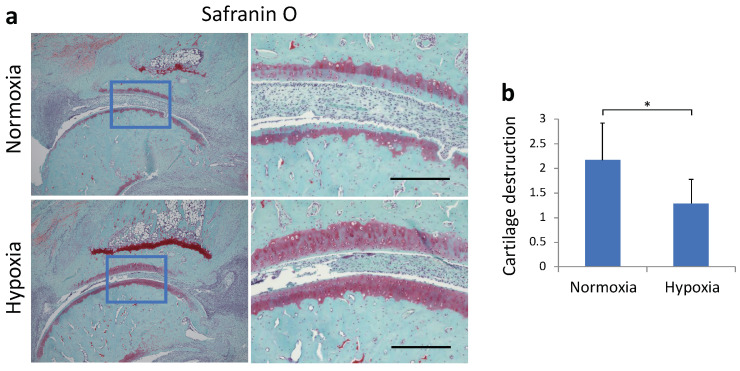
(**a**) Representative micrographs of safranin-O-stained sagittal sections of ankle joint. (**b**) The cartilage evaluation scores based on the histological score (mean ± standard deviation) are shown. Each value represents the mean ± SD (n = 5); * *p* < 0.05. Scale bar = 200 μm.

To evaluate the histological effects on the joints, we performed safranin O staining of rat ankle joints on day 28 (Figure 5a). The normoxia group showed more irregular cartilage surface, thinning of cartilage, and reduced staining of the cartilage matrix than the hypoxia group. The cartilage destruction score was also significantly lower in the hypoxia group (*p* = 0.027, Figure 5b).

## 3. Discussion

In this study, we evaluated the effects of sustained hypoxia on MH7A cells and arthritis in a rat model of CIA. In vitro, sustained hypoxia decreased the expression of HIF-1α and pro-inflammatory cytokines. In vivo, sustained hypoxia suppressed arthritis and joint destruction in the rat model of CIA compared with the normoxia group.

Cells adapt by adjusting the HIF-1α level according to the duration and degree of hypoxia [11]. Under sustained hypoxic conditions, HIF-1α protein expression generally peaks around 4–8 h and then decreases continuously [10,12,13]. This phenomenon was mainly speculated to be due to the high efficiency of HIF-1α hydroxylation by PHD under hypoxic conditions [14]. In the present study, the transcription of *HIF1A* and *EGLN1* increased in MH7A cells in a time-dependent manner. *HIF1A* expression reached equilibrium after 48 h, whereas *EGLN1* expression further increased after 48 h. The rate of increase in *EGLN1* expression was higher than that of *HIF1A* under sustained hypoxia. In addition, the protein level of HIF-1α initially increased and continuously decreased thereafter. These results suggest that HIF-1α protein is upregulated during short-term hypoxia, but is downregulated during sustained hypoxia due to degradation of the HIF-1α protein via negative feedback.

RA pathogenesis involves an inflammatory response mediated by pro-inflammatory cytokines, such as TNF-α, interleukin (IL)-1β, and IL-6, in the synovial tissue [15]. TNF-α is one of the most important pro-inflammatory cytokines involved in the pathogenesis of RA. TNF-α contributes to the pathogenesis of RA [16] via bone destruction and resorption, and promotes pannus formation due to fibroblast hyperplasia [17,18,19]. Therefore, anti-TNF-α agents exert therapeutic effects and act as biological agents that improve both inflammation and joint damage in patients with RA [20]. IL-1β, another important pro-inflammatory cytokine, is involved in synovial proliferation and cartilage destruction in RA, and is also considered as a therapeutic target [17]. In addition, in the pathogenesis of RA, a hypoxic environment is associated with the promotion of angiogenesis, pannus formation, and inflammatory processes due to abnormal activation of HIF-1α [21]. Activation of HIF-1α has been reported to induce the activation of pro-inflammatory cytokines, such as TNF-α and IL-1β [9,22,23]. In the present study, the expression of pro-inflammatory cytokines, namely, *TNFA*, *IL1B*, and *IL6*, transiently increased in MH7A cells under a short duration of hypoxia exposure (1 h). The expression of these cytokines decreased following sustained hypoxia after three hours and was predominantly decreased compared with the control at certain time points. In addition, the DFX-mediated activation of HIF-1α increased *TNFA* expression. These results suggest that HIF-1α contributes to the transcription of pro-inflammatory cytokines and that sustained hypoxia suppresses TNF-α expression by suppressing HIF-1α via negative feedback.

Reports showed that hypoxia occurring in the synovial tissue of RA correlates with the intensity of inflammatory processes and cell migration [24]. Therefore, hypoxia has traditionally been considered a factor that exacerbates arthritis by inducing HIF-1α expression in RA. However, hypoxia not only enhances HIF-1α, but also causes an HIF-α negative feedback effect. Recently, rearing of rats under 10% hypoxia for 14 days suppressed tumor progression in lung cancer via suppression of HIF-1α [25]. However, few studies have examined the relationship between a hypoxic environment and RA, although researchers have attempted to control arthritis using hypoxic stimulation prior to immunization, which is equivalent to the conditions at 3000 m altitude [26]. In the present study, a rat model of CIA was maintained under continuous hypoxia after immunization. As a result, the onset of joint swelling was delayed and the degree of joint swelling was ameliorated in the hypoxia group compared with the normoxia group. The number of swollen joints was also reduced. Furthermore, histological evaluation of the ankle joint showed less cartilage destruction in the hypoxia group than in the normoxia group. These results indicate that sustained hypoxia exerts an inhibitory effect on arthritis in the rat CIA model after immunization.

In this rat CIA model, arthritis occurs as a result of immunization to type II collagen. The observed delay in arthritis onset may have been caused by hypoxia affecting the immune response of the rat CIA model. However, it was reported that hypoxia suppresses the migration of macrophages, causing the cells to stagnate in the affected area, leading to increased local production of reactive oxygen species (ROS), which in turn enhance the immune response [27]. We therefore postulate that the RA arthritis model was successfully generated under hypoxia. Overall, we suggest that sustained hypoxia also suppresses arthritis via the negative feedback of HIF-1α. Thus, the regulation of HIF-1α expression by hypoxia may be a new treatment strategy for RA in combination with existing pharmacotherapy.

If HIF-1α is increased under transient hypoxia, one might expect it to decrease under the opposite condition, hyperoxia. However, hyperoxia was instead found to enhance the synthesis and stability of HIF-1α protein [28]. Various mechanisms have been reported to be responsible for this phenomenon, including the effect of ROS and the creation of a local hypoxic environment following vasoconstriction [29]. Thus, few studies have reported HIF-1α suppression by hyperoxia, leading us to postulate that no significant therapeutic effect would be achieved.

This study has several limitations. First, although we used the rat CIA model, we were unable to clarify the relationship between hypoxia and the immune response to type II collagen. Second, certain results were not statistically significant, including those obtained via Western blotting. Third, anti-inflammatory cytokines were not evaluated, which should be carried out in the future to validate the current study results. Finally, we were unable to evaluate HIF-1α expression at different time points in vivo. Further studies will be needed to confirm these results.

## 4. Materials and Methods

### 4.1. Preparation of MH7A

MH7A cells, in which rheumatoid fibroblast-like synoviocytes were transformed with the Simian virus 40 T antigen, were used this study. MH7A cells were provided by RIKEN BRC through the National Bio Resource Project of the MEXT/AMED, Japan. MH7A cells were cultured as monolayers in 75 cm^2^ flasks at 37 °C in the presence of 5% CO_2_ and 95% humidified air in Roswell Park Memorial Institute (RPMI) 1640 medium (Nacalai Tesque, Kyoto, Japan) supplemented with 10% fetal bovine serum (FBS; Equitech-Bio, TX, USA) and 1% penicillin–streptomycin mixed solution (Nacalai Tesque) (complete RPMI medium).

### 4.2. Cultures under Different Levels of Oxygen Tension

MH7A cells were treated with trypsin/ethylenediaminetetraacetic acid (EDTA), resuspended in complete RPMI medium, seeded in 6-well culture plates at a density of 1 × 10^6^ cells per well with 2 mL complete RPMI medium, and incubated under normoxic conditions (normoxia: 5% CO_2_, 21% O_2_, and 74% N_2_) in a Napco Model 5100 CO_2_ incubator (Wakenyaku Co. Ltd., Kyoto, Japan) at 37 °C. Twenty-four hours later, the cells were incubated for an additional 24 h under normoxic conditions or were incubated for an additional 1, 3, 6, 12, 24, and 72 h under hypoxic conditions (hypoxia: 1% O_2_, 5% CO_2_, and 94% N_2_) in a multigas incubator (MODEL 9200, Wakenyaku Co. Ltd., Kyoto, Japan).

### 4.3. Inhibition of PHD in Chronic Hypoxia

As mentioned above, MH7A cells were seeded in 6-well culture plates and incubated in complete RPMI medium under normoxia for 24 h at 37 °C. DFX (Mesylate, Sigma-Aldrich, Tokyo, Japan), a PHD inhibitor, was added to the medium at a concentration of 100 μM, and the cells were cultured for an additional 24 h under normoxia or for 1, 6, 24, and 72 h under hypoxia using the multigas incubator.

### 4.4. Real-Time Polymerase Chain Reaction (PCR)

Following incubation under normoxia or hypoxia, the cells were collected, and total RNA was extracted using ISOGEN II (Nippon Gene, Osaka, Japan). cDNA was synthesized by reverse transcription using ReverTra Ace^®^ qPCR RT master mix (Toyobo, Osaka, Japan) according to the manufacturer’s instructions. Quantitative reverse transcription PCR (qRT-PCR) was performed using a Biosystem 7300 (Applied Biosystems, Carlsbad, CA, USA) with TaqMan Assay-on-Demand gene expression primer/probe sets (Invitrogen and Applied Biosystems) for *EGLN1*, *HIF1A*, *VEGF*, *TNFA* (Hs00174128_m1), *IL1B* (Hs00174097_m1), and *IL6* (Hs00174131_m1). Each 25 µL reaction mixture contained 2 µL cDNA (100 ng) and 12.5 µL TaqMan gene expression PCR master mix (Toyobo, Osaka, Japan) for the target gene. The amplification protocol consisted of 40 cycles of denaturation at 95 °C for 15 s and annealing and extension at 60 °C for 1 min. Relative changes in gene expression were calculated using the comparative Ct method. The 18S ribosomal RNA was used as the internal control. Primers used in the study are provided in Table 1.

### 4.5. Western Blot Analysis

Cells were harvested by scraping and suspended in 150 μL radioimmunoprecipitation assay (RIPA) buffer with protease inhibitor cocktail (Nacalai Tesque, Kyoto, Japan), and solubilization was achieved within 10 min. The samples were centrifuged at 10,000× *g* for 10 min and the supernatant was aspirated. Protein concentration was estimated using a bicinchoninic acid (BCA) assay and equal amounts of total proteins were loaded onto polyacrylamide gels for sodium dodecyl sulfate polyacrylamide gel electrophoresis (SDS-PAGE). Samples containing 10 μg protein were separated on 10% Bis-Tris gel via NuPAGE^®^ electrophoresis using 5% MOPS SDS running buffer (Thermo Fisher Scientific Inc., Waltham, MA, USA). The separated proteins were wet-blotted onto a nitrocellulose membrane, which was blocked by shaking in Blocking One solution (Nacalai Tesque, Kyoto, Japan) for 60 min at 20–25 °C temperature. The blots were probed for β-actin and other primary antibodies and were washed thrice with Tris buffered saline-Tween 20 (TBST) and incubated for 60 min at 20–25 °C with peroxidase-conjugated secondary antibodies at a 1:4000 dilution. The blots were again washed thrice with TBST, and chemiluminescent signals were visualized using a Chemi Lumi One imager (Nacalai Tesque, Kyoto, Japan). Protein band intensities were assessed using the ECL Select LAS500 reagent (GE Healthcare, Chicago, IL, USA). The primary antibodies against HIF-1α (ab51608, Abcam, Cambridge, UK), PHD2 (19886-1-AP, Proteintech, Rosemont, IL, USA), and β-actin (A2228, Cell Signaling Technology, Topsfield, MA, USA), and anti-mouse IgG and anti-rabbit IgG secondary antibodies were purchased from Cell Signaling Technology.

### 4.6. CIA Model

The rat CIA model has several similarities to human RA and has been widely used for in vivo RA studies. To create this model for our study, type II collagen (CII; Collagen Research Center, Tokyo, Japan) and Freund’s incomplete adjuvant (FIA; Sigma-Aldrich, St. Louis, MO, USA) were mixed and emulsified in a 1:1 ratio on ice. The CII/FIA solution (200 μL) was injected intradermally into the base of the tail of 8 week old male Dark Agouti (DA) rats (Shimizu Laboratory Suppliers, Kyoto, Japan) (body weight: 155–180 g) [30]. The rats were housed under controlled conditions of 22–26 °C and 40–60% humidity, with a 12 h light/dark cycle. They were allowed free access to food and water and were euthanized on day 28 after commencing the experimental protocol. All animal experiments were conducted in accordance with the animal research guidelines of the Kyoto Prefectural University of Medicine, Kyoto, Japan (code no. M26-29).

### 4.7. Rearing Rats in a Hypoxic Chamber

**Figure 6:**
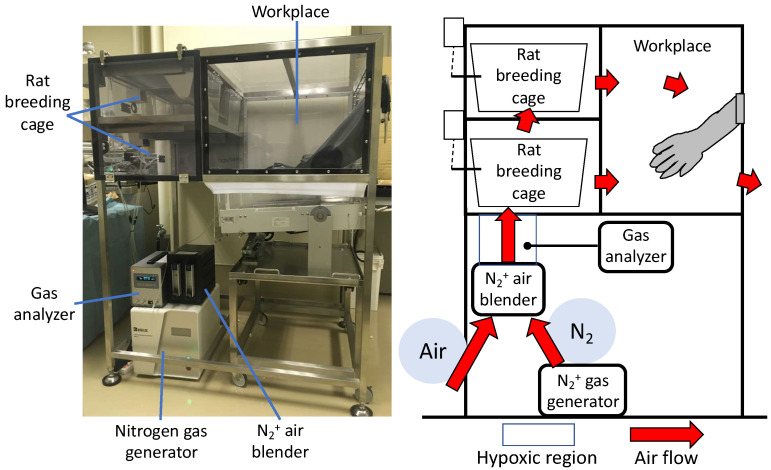
Hypoxic chamber. Nitrogen generated by the N_2_+ gas generator is mixed with the outside air using a N_2_+ air blender to generate hypoxia. The oxygen concentration in the chamber could be adjusted as desired by circulating low oxygen levels through the chamber.

A unique hypoxia chamber was fabricated (Natsume Seisakusho Co., Ltd., Wakenyaku Co., Ltd., Kyoto, Japan) in which the oxygen concentration can be arbitrarily set (Figure 6). The nitrogen generated from the N_2_+ gas generator was mixed with ambient air at an arbitrary ratio using a N_2_+ air blender to generate hypoxia. The generated hypoxic air was circulated in the rat breeding cage and workplace to create a hypoxic environment in the chamber. The oxygen concentration in any part of the chamber could be measured using a gas analyzer. The oxygen concentration in the chamber can be adjusted as desired by adjusting the N_2_+ air blender and was maintained at 4–20%. The oxygen concentration was set preliminarily, and a concentration of 12% was set as the concentration that had the least effect on the body weight and other general conditions of normal DA rats (data not shown).

### 4.8. Body Weight, Paw Volume, and Clinical Score

The rat model of CIA was reared under hypoxic conditions in a hypoxic chamber and compared with rats reared under normoxia. Body weight, paw volume, and clinical score were measured on days 0, 2, 4, 6, 8, and 10 post-immunization and every day after day 12. The paw volume was measured using a water replacement plethysmometer (Unicom Japan, Tokyo, Japan). In the hypoxia group, rats were temporarily exposed to normoxic conditions for less than 3 minutes when assessed for paw volume and other parameters.

The clinical score was defined as follows: score 0, normal paw; score 1, inflammation and swelling of one toe; score 2, inflammation and swelling of > 1 toe with inflammation and swelling of the entire paw or mild swelling of the entire paw; score 3, inflammation and swelling of the entire paw; score 4, severe inflammation and swelling of the entire paw or ankylosed paw [31].

### 4.9. Histochemical Analysis

The right ankle joints of rats were removed and fixed in 4% paraformaldehyde (Wako, Osaka, Japan). They were then decalcified with 20% EDTA and embedded in paraffin. The center of each ankle joint was sliced into 6 µm thick sagittal slices and stained with safranin O. Arthritic changes, such as infiltration of inflammatory cells, synovial proliferation, destruction of articular cartilage, and bone erosion, were then evaluated histologically, and cartilage destruction was measured on a score of 0–3, from no damage to completely destroyed cartilage layers, as described by Weinberger et al. [32].

### 4.10. Statistical Analysis

All experimental data are presented as mean ± standard deviation (SD). Parametric one-way analysis of variance (ANOVA) was used to examine statistical differences among the groups. The Tukey–Kramer test was used to determine the specific differences between the groups if the results were significant. In all analyses, *p* < 0.05 was considered to indicate a statistically significant difference.

## 5. Conclusions

Our study suggests, for the first time, that sustained hypoxia suppresses HIF-1α and pro-inflammatory cytokine expression in MH7A cells, while inhibiting arthritis in a CIA rat model. Low-impact sustained hypoxia effectively delayed the onset of joint swelling, suppressed arthritis, and ameliorated cartilage pathological changes via negative feedback of HIF-1α. Hence, if HIF-1α expression can be regulated via the oxygen level in the environment, it may be used as a new mode of treatment with safe and potent effects in high-risk RA patients, in combination with existing drug-based therapies.

## Figures and Tables

**Table 1:** The primer sequences for real-time PCR.

Gene		Sequence
18S ribosomal RNA	forward reverse probe	5′-ATGAGTCCACTTTAAATCCTTTAACGA-3′ 5′-CTTTAATATACGCTATTGGAGCTGGAA-3′ 5′-(FAM) ATCCATTGGAGGGCAAGTCTGGTGC (BHQ)-3′
*EGLN1*	forward reverse	5′-CGACCTGATACGCCACTGT-3′ 5′-GTTCCATTGCCCGGATAAC-3′
*HIF1A*	forward reverse	5′-TTTTCAAGCAGTAGGAATTGGAA-3′ 5′-GTGATGTAGTAGCTGCATGATCG-3′
*VEGF*	forward reverse	5′-GCAGCTTGAGTTAAACGAACG-3′ 5′-GGTTCCCGAAACCCTGAG-3′

## Data Availability

Not applicable.

## References

[1] McInnes I.B., Schett G. (2011). The pathogenesis of rheumatoid arthritis. N. Engl. J. Med..

[2] Comella N.F.L., Matilla M.F., Cuesta J.A.C. (2016). Have complementary therapies demonstrated effectiveness in rheumatoid arthritis?. Reumatol. Clin..

[3] Singh J.A., Wells G.A., Christensen R., Ghogomu E.T., Maxwell L., Macdonald J.K., Filippini G., Skoetz N., Francis D., Lopes L.C. (2011). Adverse effects of biologics: A network meta-analysis and Cochrane overview. Cochrane Database Syst. Rev..

[4] Shimomura S., Inoue H., Arai Y., Nakagawa S., Fujii Y., Kishida T., Ichimaru S., Tsuchida S., Shirai T., Ikoma K. (2018). Treadmill running ameliorates destruction of articular cartilage and subchondral bone, not only synovitis, in a rheumatoid arthritis rat model. Int. J. Mol. Sci..

[5] Lund-Olesen K. (1970). Oxygen tension in synovial fluids. Arthritis Rheum..

[6] Bruick R.K., McKnight S.L. (2001). A conserved family of prolyl-4-hydroxylases that modify HIF. Science.

[7] Koh M.Y., Powis G. (2012). Passing the baton: The HIF switch. Trends Biochem. Sci..

[8] Yao L., Kan E.M., Lu J., Hao A., Dheen S.T., Kaur C., Ling E.A. (2013). Toll-like receptor 4 mediates microglial activation and production of inflammatory mediators in neonatal rat brain following hypoxia: Role of TLR4 in hypoxic microglia. J. Neuroinflamm..

[9] Gao X., Li Y., Wang H., Li C., Ding J. (2017). Inhibition of HIF-1α decreases expression of pro-inflammatory IL-6 and TNF-α in diabetic retinopathy. Acta Ophthalmol..

[10] Uchida T., Rossignol F., Matthay M.A., Mounier R., Couette S., Clottes E., Clerici C. (2004). Prolonged hypoxia differentially regulates hypoxia-inducible factor (HIF)-1 alpha and HIF-2 alpha expression in lung epithelial cells: Implications of natural antisense HIF-1 alpha. J. Biol. Chem..

[11] Saxena K., Jolly M.K. (2019). Acute vs. chronic vs. cyclic hypoxia: Their differential dynamics, molecular mechanisms, and effects on tumor progression. Biomolecules.

[12] Bartoszewski R., Moszynska A., Serocki M., Cabaj A., Polten A., Ochocka R., Dell’Italia L., Bartoszewska S., Kroliczewski J., Dabrowski M. (2019). Primary endothelial cell-specific regulation of hypoxia-inducible factor (HIF)-1 and HIF-2 and their target gene expression profiles during hypoxia. FASEB J..

[13] Lin Q., Cong X., Yun Z. (2011). Differential hypoxic regulation of hypoxia-inducible factors 1 alpha and 2 alpha. Mol. Cancer Res..

[14] Holmquist-Mengelbier L., Fredlund E., Lofstedt T., Noguera R., Navarro S., Nilsson H., Pietras A., Vallon-Christersson J., Borg A., Gradin K. (2006). Recruitment of HIF-1α and HIF-2α to common target genes is differentially regulated in neuroblastoma: HIF-2α promotes an aggressive phenotype. Cancer Cell.

[15] Boissier M.C. (2011). Cell and cytokine imbalances in rheumatoid synovitis. Jt. Bone Spine.

[16] Brennan F.M., McInnes I.B. (2008). Evidence that cytokines play a role in rheumatoid arthritis. J. Clin. Investig..

[17] Zwerina J., Hayer S., Tohidast-Akrad M., Bergmeister H., Redlich K., Feige U., Dunstan C., Kollias G., Steiner G., Smolen J. (2004). Single and combined inhibition of tumor necrosis factor, interleukin-1, and RANKL pathways in tumor necrosis factor-induced arthritis: Effects on synovial inflammation, bone erosion, and cartilage destruction. Arthritis Rheum..

[18] Williams R.O., Inglis J.J., Simelyte E., Criado G., Sumariwalla P.F. (2005). Analysing the effect of novel therapies on cytokine expression in experimental arthritis. Int. J. Exp. Pathol..

[19] Fujii Y., Inoue H., Arai Y., Shimomura S., Nakagawa S., Kishida T., Tsuchida S., Kamada Y., Kaihara K., Shirai T. (2019). Treadmill running in established phase arthritis inhibits joint destruction in rat rheumatoid arthritis models. Int. J. Mol. Sci..

[20] Mitoma H., Horiuchi T., Tsukamoto H., Ueda N. (2018). Molecular mechanisms of action of anti-TNF-α agents—Comparison among therapeutic TNF-α antagonists. Cytokine.

[21] Giatromanolaki A., Sivridis E., Maltezos E., Athanassou N., Papazoglou D., Gatter K.C., Harris A.L., Koukourakis M.I. (2003). Upregulated hypoxia inducible factor-1 alpha and -2 alpha pathway in rheumatoid arthritis and osteoarthritis. Arthritis Res. Ther..

[22] Yamaguchi R., Kamiya N., Adapala N.S., Drissi H., Kim H.K. (2016). HIF-1-dependent IL-6 activation in articular chondrocytes initiating synovitis in femoral head ischemic osteonecrosis. J. Bone Jt. Surg..

[23] Xing J., Lu J. (2016). HIF-1 alpha activation attenuates IL-6 and TNF-alpha pathways in hippocampus of rats following transient global ischemia. Cell Physiol. Biochem..

[24] Ng C.T., Biniecka M., Kennedy A., McCormick J., Fitzgerald O., Bresnihan B., Buggy D., Taylor C.T., O’Sullivan J., Fearon U. (2010). Synovial tissue hypoxia and inflammation in vivo. Ann. Rheum. Dis..

[25] Reiterer M., Colaço R., Emrouznejad P., Jensen A., Rundqvist H., Johnson R.S., Branco C. (2019). Acute and chronic hypoxia differentially predispose lungs for metastases. Sci. Rep..

[26] Shi M., Cui F., Liu A.J., Ma H.J., Cheng M., Song S.X., Yuan F., Li D.P., Zhang Y. (2015). The protective effects of chronic intermittent hypobaric hypoxia pretreatment against collagen-induced arthritis in rats. J. Inflamm..

[27] Tan H.Y., Wang N., Li S., Hong M., Wang X., Feng Y. (2016). The reactive oxygen species in macrophage polarization: Reflecting its dual role in progression and treatment of human diseases. Oxid. Med. Cell Longev..

[28] Catrina S.B., Okamoto K., Pereira T., Brismar K., Poellinger L. (2004). Hyperglycemia regulates hypoxia-inducible Factor-1α Protein Stability and function. Diabetes.

[29] Terraneo L., Virgili E., Caretti A., Bianciardi P., Samaja M. (2014). In vivo hyperoxia induces hypoxia-inducible factor-1alpha overexpression in LNCaP tumors without affecting the tumor growth rate. Int. J. Biochem. Cell Biol..

[30] Bakharevski O., Stein-Oakley A.N., Thomson N.M., Ryan P.F. (1998). Collagen induced arthritis in rats. Contrasting effect of subcutaneous versus intradermal inoculation of type II collagen. J. Rheumatol..

[31] Jin H., Ma N., Li X., Kang M., Guo M., Song L. (2019). Application of GC/MS-based metabonomic profiling in studying the therapeutic effects of *Aconitum carmichaeli* with *Ampelopsis japonica* extract on collagen-induced arthritis in rats. Molecules.

[32] Weinberger A., Halpern M., Zahalka M.A., Quintana F., Traub L., Moroz C. (2003). Placental immunomodulator ferritin, a novel immunoregulator, suppresses experimental arthritis. Arthritis Rheum..

